# VTC-Net: A Semantic Segmentation Network for Ore Particles Integrating Transformer and Convolutional Block Attention Module (CBAM)

**DOI:** 10.3390/s26030787

**Published:** 2026-01-24

**Authors:** Yijing Wu, Weinong Liang, Jiandong Fang, Chunxia Zhou, Xiaolu Sun

**Affiliations:** 1 College of Electric Power, Inner Mongolia University of Technology, Hohhot 010051, China; wuyijing188@163.com; 2 China-Mongolia Belt and Road Joint Laboratory of Mineral Processing Technology, Inner Mongolia Academy of Science and Technology, Hohhot 010000, China; liangwn1@163.com; 3 Inner Mongolia Key Laboratory of Intelligent Perception and System Engineering, Hohhot 010080, China; zhoull@imut.edu.cn

**Keywords:** ore image segmentation, machine vision, semantic segmentation, transformer, attention mechanism

## Abstract

In mineral processing, visual-based online particle size analysis systems depend on high-precision image segmentation to accurately quantify ore particle size distribution, thereby optimizing crushing and sorting operations. However, due to multi-scale variations, severe adhesion, and occlusion within ore particle clusters, existing segmentation models often exhibit undersegmentation and misclassification, leading to blurred boundaries and limited generalization. To address these challenges, this paper proposes a novel semantic segmentation model named VTC-Net. The model employs VGG16 as the backbone encoder, integrates Transformer modules in deeper layers to capture global contextual dependencies, and incorporates a Convolutional Block Attention Module (CBAM) at the fourth stage to enhance focus on critical regions such as adhesion edges. BatchNorm layers are used to stabilize training. Experiments on ore image datasets show that VTC-Net outperforms mainstream models such as UNet and DeepLabV3 in key metrics, including MIoU (89.90%) and pixel accuracy (96.80%). Ablation studies confirm the effectiveness and complementary role of each module. Visual analysis further demonstrates that the model identifies ore contours and adhesion areas more accurately, significantly improving segmentation robustness and precision under complex operational conditions.

## 1. Introduction

In mineral processing, the particle size distribution of feed materials and products is a key indicator for assessing ore processability. It also provides critical guidance for adjusting operational parameters in crushing, screening, and sorting circuits [1,2,3]. Visual-based online particle size analysis systems must first segment individual particles from conveyor belt images to derive size distributions, where the accuracy of segmentation directly determines the reliability of subsequent statistical analysis. However, inherent particle adhesion and occlusion within ore particle clusters often lead to blurred or indistinct boundaries, which remains a primary constraint on the precision of visual inspection systems [4,5,6].

Traditional image processing methods for ore segmentation mainly include thresholding, region-based analysis, edge detection, and watershed algorithms [7,8,9]. While effective for images with clear particle contours and simple backgrounds, these methods demonstrate limited accuracy when dealing with complex, adhering, and overlapping ore particle clusters. With advances in deep learning, convolutional neural network-based segmentation has become the mainstream approach. Representative architectures such as FCN, UNet, and DeepLabV3, leveraging their powerful multi-scale feature extraction capabilities, have significantly improved segmentation performance [10,11,12]. To further address the challenges of adhesion and stacking, researchers have proposed various enhanced algorithms. For example, Li et al. [13] introduced a two-stage detection-guided segmentation framework (Det-SAM-Ore), which locates ore particles, generates bounding boxes, and feeds them into the Segment Anything Model (SAM) to achieve precise segmentation, improving efficiency across multiple ore types. Yang et al. [14] increased model sensitivity to ore boundaries by incorporating contour-aware loss functions and using a pre-trained VGG16 as the encoder. Deo et al. [15] proposed an improved UNet with normalization layers and 1 × 1 convolutional modules to reduce computational complexity, effectively enhancing real-time performance and accuracy in segmenting iron ore pellets for size analysis. Wang et al. [16] developed a lightweight ReUNet model that showed strong performance across several public datasets. Collectively, these studies have focused on edge precision, multi-scale feature fusion, and model lightweighting. Nevertheless, many models still tend to prioritize larger ore particles, often overlooking finer particles that exhibit severe adhesion, particularly in datasets rich in small particle sizes.

To address the complexities of real-world industrial scenarios, Fu et al. [17] proposed integrating Simple Linear Iterative Clustering (SLIC) with UNet, treating ore particle contours as an independent category for three-class segmentation. This method demonstrated superior performance over traditional watershed algorithms on industrial conveyor belts. Wang et al. [18] developed MSBA-UNet, which combines multi-scale connectivity and boundary awareness, utilizing convex hull defect detection to separate deeply concave adhered particles and achieving precise classification of boundary pixels. Liu et al. [19] designed a two-stage network that first obtains preliminary segmentation results using UNet and then refines them with a self-training network to enhance accuracy. Zhang et al. [20] introduced OIS-Net for conveyor belt ore image segmentation, effectively improving feature fusion and showing good performance on single-type ore datasets. Although these studies partially account for practical transportation conditions, most enhanced methods still struggle with multi-scale, heavily adhered ore clusters exhibiting high textural similarity.

In mineral processing, particle clusters consist of ores with diverse sizes and compositions. Significant size variations often cause fine particle features to be overlooked, while inter-particle adhesion, shared boundaries, and similar gray scale values further complicate segmentation [21,22,23,24,25]. As a result, existing models show limited generalization under complex conditions, frequently leading to undersegmentation (merging of adjacent particles) and misjudgment (incorrect identification of non-ore regions). To overcome these challenges, this study proposes a semantic segmentation model for ore particles that integrates Transformer, BatchNorm, and CBAM attention mechanisms, aiming to improve segmentation accuracy across multi-scale ore particle clusters.

## 2. Methodology for Image Data

### 2.1. Datasets Collection

**Figure 1:**
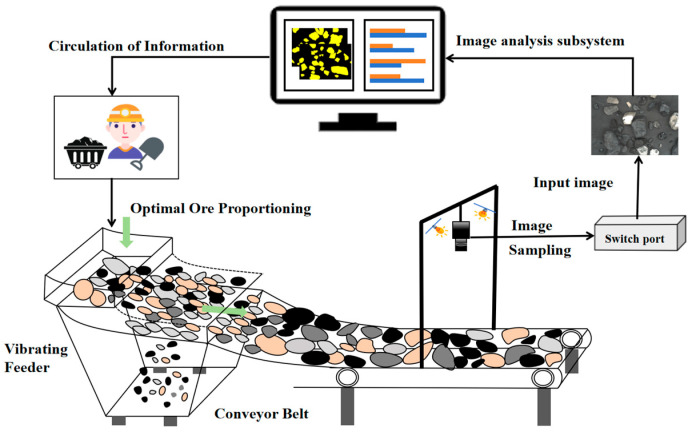
Detailed implementation flowchart.

**Figure 2:**
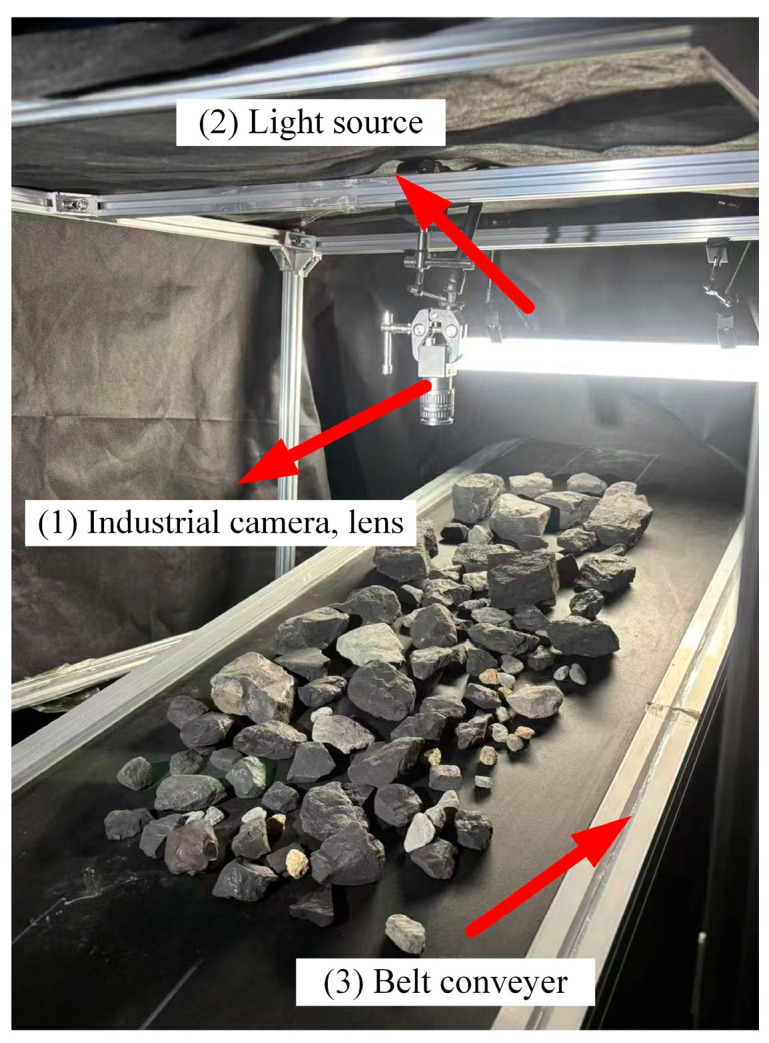
Schematic diagram of the image acquisition platform.

In mineral processing, a typical setup for visual-based particle size detection is illustrated in Figure 1. Ore material is first uniformly distributed onto a conveyor belt via a feeder or vibrating screen. An industrial camera, mounted above the belt, captures real-time images of the material flow. After preprocessing, these images are input into a trained segmentation model to perform online particle size recognition. The detection results are instantly fed back to the control system, providing operational guidance for subsequent sorting or for adjusting preceding crushing and screening stages [26]. To replicate actual working conditions, a laboratory image acquisition platform was established, as shown in Figure 2. The platform consists of a belt conveyor, an industrial camera (HIKVISION MV-CA004, Hangzhou, China), a lens (HIKVISION ZX-SF1214B, Hangzhou, China), and a linear light source (KOMAVISION KM-2BRD6020, Shenzhen, China) [27]. The experiments focused on raw coal fed into coal preparation processes, with particle sizes ranging from 13 mm to 100 mm. It should be noted that while the proposed VTC-Net model framework is designed for broad application in mineral processing (including coal, metallic, and non-metallic ores), the experimental validation and performance analysis presented in this paper are based specifically on raw coal (13–100 mm particle size) as the experimental validation material. Therefore, in the subsequent sections detailing the experiments, we will consistently use ‘raw coal’ to refer to the target material.

### 2.2. Data Preprocessing

To ensure the quality of the dataset, invalid images were removed and 500 images (resolution 720 × 540 pixels) were ultimately retained. Image annotation was performed using the Labelme (version 4.5.12) tool. For ambiguous particle boundaries, the point of maximum gradient change was marked by the annotators. Adhering particles with no visible separation were labeled as a single object, which aligns with the practical requirements of particle size analysis. A binary segmentation framework was adopted, as the core objective is to segment raw coal particles from the industrial background. Consequently, each pixel was assigned one of two mutually exclusive labels: ‘raw coal’ or ‘background’. For the evaluation metrics (e.g., MIoU, MPA), the total number of classes *C* is therefore 2. This labeling strategy directly supports the goal of accurately identifying and delineating individual particles on the conveyor belt for subsequent size analysis.

**Figure 3:**
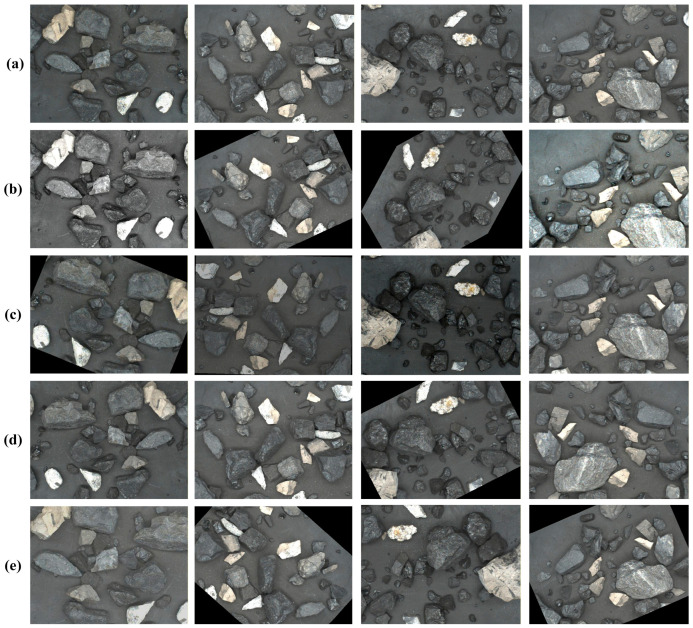
Image augmentation. (**a**) Original image samples. (**b**–**e**) Enhanced image samples.

To enhance the model’s generalization capability, data augmentation techniques [28,29] were employed to expand the dataset, including color enhancement (0.5–1.5), random rotation (0–45°), flipping and cropping (0.8–1.2). To simulate lighting variations and changes in camera angles that may occur in real-world industrial environments, four enhancement techniques were randomly applied to each image during the process, with multiple methods allowed to be combined on the same image. As illustrated in Figure 3, this process increased the total number of images to four times the original size. The dataset was then split into training, validation, and test sets in an 8:1:1 ratio. Furthermore, all original images were resized to 512 × 512 pixels via bilinear interpolation [30] to accelerate model training.

## 3. Methods

### 3.1. VTC-Net Architecture

**Figure 4:**
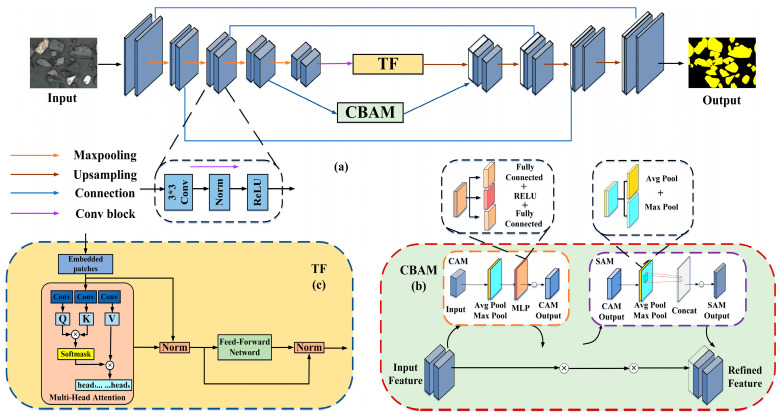
(**a**) VTC-Net network architecture diagram. (**b**) CBAM architecture diagram. (**c**) Transformer architecture diagram.

The architecture of the proposed VTC-Net is illustrated in Figure 4. The encoder employs VGG16 as its backbone for feature extraction. Each stage of the encoder consists of a convolutional block followed by a downsampling layer. The convolutional block uses 3 × 3 convolutions (stride = 1, padding = 1), each paired with a ReLU activation function and a BatchNorm layer. The BatchNorm layers standardize the inputs across the network during training, stabilizing gradient flow in deep feature learning and improving training efficiency. Downsampling is performed via 2 × 2 max-pooling layers (stride = 2), which reduce spatial resolution while expanding the receptive field to capture more abstract, global features.

To strengthen the model’s representational capacity, a Transformer module is integrated into the deepest layer of the backbone to model long-range contextual dependencies. Additionally, a Convolutional Block Attention Module (CBAM) is incorporated at the fourth feature stage to enhance focus on target raw coal regions within complex, mixed particle clusters.

The decoder reconstructs the segmented raw coal image through progressive upsampling and skip connections. At each decoder level, the input feature map is first upsampled via transposed convolution. It is then concatenated with the corresponding feature map from the encoder via skip connections, thereby preserving both high-level semantic information and low-level edge details. The fused features are subsequently refined through convolutional layers with progressively reduced channel dimensions (512, 256, 128, 64), ultimately producing a high-resolution segmentation map of the coal particles [31,32,33].

#### 3.1.1. CBAM Attention Mechanism

To enhance the model’s capacity to recognize critical raw coal particle regions and weak boundaries, a Convolutional Block Attention Module (CBAM) is incorporated into the fourth encoder stage. This module adaptively generates channel-wise and spatial attention weights, enabling the network to concentrate on key features such as particle edges and adhesion zones while effectively suppressing interference from conveyor belt background noise. Consequently, segmentation accuracy under complex adhesive conditions is improved. As illustrated in Figure 4b, the CBAM comprises an input layer, a channel attention module, a spatial attention module, and an output layer [34]. Its computational procedure is as follows:

In the channel attention stage, the input $F\in R^{C*H*W}$ feature map undergoes max pooling and average pooling operations in spatial dimensions, respectively, generating two channel description vectors to reflect each channel’s response intensity in the global space.

(1) $$F_{avg}^{c}=AvgPool(F)\in R^{C*1*1}$$

(2) $$F_{max}^{c}=MaxPool(F)\in R^{C*1*1}$$

The two vectors $F_{avg}^{c},{\;F}_{max}^{c}$ are then fed into the shared two-layer *MLP* for mapping, with the Sigmoid function generating attention weights for each channel.

(3) $$M_{c}\left(F\right)=\sigma \left({MLP(F}_{avg}^{c}){+MLP(F}_{max}^{c})\right)$$

where σ(·) denotes the Sigmoid function, which generates channel attention weights $M_{c}\left(F\right)\in R^{C*1*1}$.

Finally, the generated channel attention weights are channel-wise multiplied $F^{\prime }$, with the original feature map to enhance key channel information.

(4) $$F^{\prime }=M_{c}\left(F\right)⊙F$$

In the spatial attention stage, the feature maps enhanced $F^{\prime }$ by channel attention undergo max pooling and mean pooling along the channel dimensions, generating two-dimensional spatial representations that, respectively, capture maximum and average responses.

(5) $$F_{avg}^{s}={AvgPool}_{c}(F^{\prime })\in R^{1*H*W}$$

(6) $$F_{max}^{s}={MaxPool}_{c}(F^{\prime })\in R^{1*H*W}$$

Subsequently, the two spatial maps are spliced along the channel dimension, and feature fusion is performed using a 7 × 7 convolution kernel to generate attention weight maps for each spatial position.

(7) $$M_{s}\left(F^{\prime }\right)=\sigma ({Conv}_{7*7}([F_{avg}^{s};F_{max}^{s}]))$$

Finally, the spatial attention and $M_{s}\left(F^{\prime }\right)\in R^{1*H*W}$ channel-enhanced feature map are multiplied with the input feature to enhance key spatial positions, thereby suppressing background interference and highlighting the raw coal target region in the feature map.

(8) $${F^{\prime \prime }=M}_{s}\left(F^{\prime }\right)⊙F^{\prime }$$

Through this two-stage attention mechanism, the CBAM output feature map $F^{\prime \prime }$ is significantly enhanced in both the channel and spatial dimensions. This enhancement enables the network to more accurately highlight target ore regions when processing mixed ore particles of varying sizes, thereby mitigating the adverse effects of particle adhesion and multi-scale variations.

#### 3.1.2. Transformer Blocks

As shown in Figure 4, a Transformer module is integrated into the deepest layer of the backbone network to address the inherent characteristics of multi-granular ore images. Utilizing a multi-head self-attention mechanism, the module establishes long-range dependencies across different regions of the image and enhances multi-scale information interaction through feature fusion. This design compensates for a key limitation of purely convolutional architectures—their local receptive fields, which often fail to capture relationships between distant ore particles within the same image. By enabling the simultaneous integration of local texture details and global contextual cues, the model’s ability to represent complex spatial structures is substantially improved [35,36]. Figure 4c illustrates the architecture of the Transformer module, which processes the input feature map through the following computational steps:

First, the input feature map $F\in R^{C*H*W}$ is divided into a sequence of N=H×W non-overlapping patches. Each patch is linearly projected into a d-dimensional vector $X\in R^{N\times d}$. A learnable position embedding $E_{pos}\in R^{N\times d}$ is then added to the sequence, resulting in the input representation $X_{i}=X+E_{pos}$.

For the input feature $X_{i}$, trainable weight matrices $W^{Q},{\;W}^{K}$, $W^{V}\in R^{d\times d_{k}}$ are used to map it into query (Q), key (K), and value (V) matrices, respectively.

(9) $$Q=X_{i}W^{Q},K=X_{i}W^{K},V={X_{i}W}^{V}$$

where $Q,K,V\in R^{N\times d_{k}},d_{k}$ represent the single-head attention dimension for the calculation of attention weights as follows.

(10) $$Attention\left(Q,K,V\right)=softmax(\frac{QK^{T}}{\sqrt{d_{k}}})V$$

The outputs of multiple attention heads are computed in parallel, concatenated, and then projected through the linear transformation $W^{O}$ to obtain the multi-head self-attention output ${MHSA(X}_{i})$. This output is then added to the original input via a residual connection, followed by layer normalization, yielding the intermediate representation Y after the *MHSA* stage.

(11) $${MHSA(X}_{i})=Concat({head}_{1},\ldots \ldots {head}_{h})W^{O}$$

(12) $$Y=Norm(X_{i}+{MHSA(X}_{i}))$$

The resulting representation Y is then passed through a two-layer feedforward neural network (*FFN*) for nonlinear transformation. Subsequently, a residual connection is applied, followed by another layer normalization operation, producing the final output Z. This output effectively integrates both global contextual information and non-local structural features.

(13) $$FFN\left(Y\right)=W_{2}\cdot GELU(W_{1}\cdot Y)$$

(14) Z=Norm(Y+FFN(Y))

where $W_{1}$ and $W_{2}$ are the weight matrices for the first and second fully connected layers.

### 3.2. Parameter Settings

On the hardware side, the experimental platform was equipped with an Intel^®^ Core™ i9-10900X CPU and an NVIDIA GeForce RTX A5000 GPU, along with 32 GB of RAM. For the software environment, the model was developed using TensorFlow 2.5 and Python 3.8, with CUDA 11.4 and cuDNN 8.2.2 configured to enable GPU-accelerated computing. The detailed hyperparameter settings of the model are provided in Table 1.

### 3.3. Loss Function

The improved Focal Loss and Dice Loss were combined to form a joint loss function system, as expressed in Equations (15) and (16). During model training, Dice Loss primarily ensures precise segmentation between raw coal and background regions, preserving the contour integrity of coal particles. Meanwhile, Focal Loss focuses on hard-to-distinguish samples by down-weighting the contribution of easy examples, thereby enhancing the model’s robustness against class imbalance and ambiguous boundaries. Specifically, this is achieved by adjusting weights for different samples by α and implementing a dynamic weight mechanism through γ. The coordinated use of these two losses significantly improves the model’s ability to delineate indistinct raw coal boundaries and adhesion regions under complex imaging conditions [37].

(15) $$Dice\;Loss=1-\frac{2\sum_{i=1}^{N}\sum_{c=1}^{C}y_{ic}{\hat{y}}_{ic}}{\sum_{i=1}^{N}\sum_{c=1}^{C}y_{ic}+\sum_{i=1}^{N}\sum_{c=1}^{C}{\hat{y}}_{ic}}$$

(16) $$Focal\;Loss=-\alpha {\left(1-p_{i}\right)}^{\gamma }log(p_{i})$$

where *N* refers to the total number of samples, *C* denotes the total number of classes, the true label of sample *i* in class *c* is represented by $y_{ic}$, while the predicted probability of sample *i* in class *c* by the model is indicated by ${\hat{y}}_{ic}$. α is the class balancing factor in the Focal Loss to address the imbalance between positive and negative samples. The probability of sample *i* being predicted as positive is denoted by $p_{i}$ and the Focal Loss focus parameter is specified by γ.

### 3.4. Evaluation Indicators

To quantitatively evaluate the performance of the segmentation model, a set of standard metrics is employed, with Mean Intersection over Union (MIoU), Mean Pixel Accuracy (MPA), and overall Accuracy serving as the primary evaluation criteria [38,39], as detailed in Equations (17)–(19).

(17) $$MIoU=\frac{1}{N}\sum_{i=1}^{N}\frac{TP_{i}}{TP_{i}+FP_{i}+FN_{i}}$$

Here, N represents the total number of classes; $TP_{i}$ denotes the number of correctly classified pixels for a certain class; $FP_{i}$ indicates the number of pixels falsely predicted as this class; and $FP_{i}$ refers to the number of pixels that actually belong to this class but are incorrectly predicted as other classes. A higher MIoU value reflects better segmentation consistency across all classes.

(18) $$MPA=\frac{1}{N}\sum_{i=1}^{N}\frac{TP_{i}}{TP_{i}+FP_{i}}$$

Here, N represents the total number of classes. A higher MPA value indicates more stable pixel classification accuracy within each category.

(19) $$Accuracy=\sum_{i=1}^{N}\frac{TP_{i}+TN_{i}}{TP_{i}+TN_{i}+FP_{i}+FN_{i}}$$

$TN_{i}$ is the number of negative classes correctly predicted as negative. The higher the Accuracy value, the stronger the model’s ability to distinguish between positive and negative classes.

By integrating MIoU, MPA, and Accuracy, a multidimensional evaluation framework is established, enabling a holistic assessment of semantic segmentation models in terms of segmentation consistency, within-class accuracy, and overall predictive performance. This comprehensive approach offers precise, actionable insights for subsequent model optimization.

### 3.5. Cross-Validation Experiments

To evaluate the model’s stability and generalization capability, a five-fold cross-validation strategy was employed. Specifically, the training and validation sets were combined into a single dataset (a total of 1800 images), which was then evenly partitioned into five subsets. In each round of experimentation, four subsets were used as the training set, while the remaining subset served as the validation set. The model was trained following the same procedure in each round, and performance was evaluated on the corresponding validation set using metrics including MIoU, MPA, and Accuracy. The mean, standard deviation, and variance of these metrics were then computed. This approach effectively mitigates the randomness inherent in single-round data partitioning, providing a more reliable assessment of the model’s performance. Experimental results are presented in Table 2.

As shown in Table 2, the average MIoU under five-fold cross-validation was 84.67% with a standard deviation of 0.64%. The small fluctuation range and low standard deviation of MIoU indicate that the model exhibits good stability under different data partitioning conditions. Additionally, the standard deviations for MPA and Acc were 0.70% and 0.15%, respectively, further validating the model’s stable performance. Based on the cross-validation results, the model demonstrating optimal performance on the validation set was selected for final evaluation on the test set. The performance comparison of the optimal model on the validation and test sets is presented in Table 3.

## 4. Discussion of the Results

### 4.1. Discussion on the Position of Adding CBAM

This section investigates the impact of integrating the CBAM attention module at different network stages by inserting it individually into the Feat1, Feat2, Feat3, and Feat4 layers. The corresponding prediction accuracies are summarized in Table 4.

Experimental results indicate that the placement of the CBAM module significantly affects model performance, with the deepest feature extraction stage yielding the best outcomes. Introducing CBAM solely at the Feat4 layer achieved the highest MIoU (88.30%), substantially outperforming placements at shallower layers such as Feat1 (85.80%) and Feat2 (85.95%). This discrepancy arises from the scale and semantic characteristics of the feature maps at each stage. In the Feat1 and Feat2 stages (with resolutions reduced to 256 × 256 and 128 × 128, respectively), features primarily contain low-level information such as texture and noise, while stable semantic structures are still underdeveloped. Applying CBAM at these stages tends to direct attention to irrelevant local details, which may interfere with the learning of deeper, more discriminative representations. In contrast, features at the Feat3 and Feat4 stages exhibit stronger semantic expression and retain more meaningful spatial structures. Here, CBAM can effectively highlight critical regions—such as adhesion boundaries between particles—thereby significantly improving segmentation accuracy. Notably, combining CBAM across multiple layers did not improve performance and even slightly underperformed compared to using it only at Feat4. This suggests that a single, strategically placed attention module in the deepest feature layer is sufficient, while adding more may introduce redundancy without gains. Therefore, deploying CBAM exclusively in the deepest layer (Feat4) represents an optimal strategy for balancing accuracy and computational efficiency.

### 4.2. Attention Mechanism Comparison Experiment

Different attention mechanism modules were integrated into the fourth layer of the backbone network, and their performance on the multi-granular coal validation set is shown in Table 5. It can be observed that the CBAM attention mechanism achieves the best result, with an MIoU of 88.30%, outperforming single dimensional attention mechanisms such as SE and ECA. This confirms the effectiveness of combining both channel and spatial attention. Compared with the CA mechanism, which also employs dual dimensional attention, the serial structure of CBAM yields slightly superior performance. These results indicate that in deeper feature layers, CBAM’s sequential filtering strategy can more accurately capture key details such as adhesion boundaries between coal particles, achieving an optimal balance between performance and efficiency.

### 4.3. Ablation Experiments

In the ablation experiments, we compared the effects of Transformer module, BatchNorm module, and CBAM module on model performance. The results are presented in Table 6.

Based on the ablation experiments results presented in Table 6, the Transformer module, the CBAM module, and the BatchNorm module all contribute to improving the performance of the base UNet model, with the first two exhibiting complementary effects. Introducing the Transformer module alone increases the MIoU by 2.29%, which surpasses the gain of 0.34% achieved by adding only the CBAM module. This indicates that global context modeling contributes more substantially to segmentation accuracy. When both modules are combined, the MIoU further rises to 88.73%, demonstrating the synergistic effect of the dual attention mechanism. Ultimately, VTC-Net achieves the best performance with an MIoU of 89.90%, confirming the effectiveness and necessity of the proposed architecture.

### 4.4. Comparative Experiments

The VTC-Net model was compared with classical networks including UNet, DeepLabV3, PSPNet, PSANet, SegFormer, and SETR. UNet employs special skip connections to enhance the model’s ability to recover object edges and details [40]. DeepLabV3 introduces multi-scale hollow convolution modules to improve multi-scale object recognition and segmentation accuracy [41]. PSPNet utilizes pyramid pooling structures to fully leverage global contextual information [42]. PSANet achieves effective fusion of shallow and deep features through parallel self-attention mechanisms [43]. SegFormer employs a lightweight Transformer architecture to capture both global and local features, thereby enhancing multi-scale segmentation capability [44]. SETR enhances segmentation performance across various scenarios by partitioning images into sequences and modeling global information through self-attention mechanisms [45]. All comparison algorithms were trained under identical conditions as VTC-Net. Table 7 presents the accuracy, intersection–union ratio, and pixel precision of each network.

Experimental results demonstrate that VTC-Net outperforms mainstream segmentation networks on multi-granularity raw coal datasets, achieving the highest Mean Intersection Union (MIoU) score while delivering optimal performance in both Mean Pixel Accuracy (MPA) and Classification Accuracy (Acc). Although VTC-Net is not optimal in terms of model parameters (Params), computational complexity (GFLOPs), and single-frame inference speed (Inference Speed). However, for the specific task of online calculation of ore particle size distribution during transportation, real-time performance does not require millisecond-level response times. Instead, it prioritizes minute-level statistical accuracy, with segmentation precision being of greater importance. The improvements in VTC-Net’s MIoU, MPA, and Acc metrics make it more suitable for this application scenario. These findings validate the effectiveness and superiority of the proposed architecture for complex coal particle segmentation tasks.

**Figure 5:**
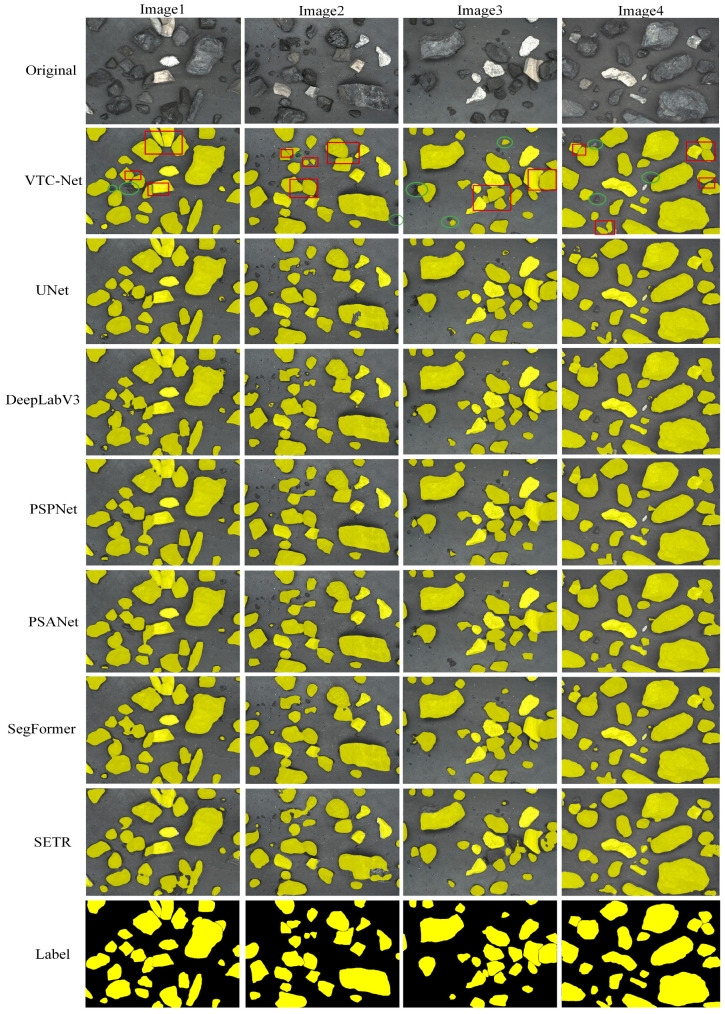
Comparison of qualitative results between VTC-Net and other mainstream networks in raw coal segmentation tasks.

Figure 5 presents the segmentation results of four sample images processed by different models. Compared to UNet, DeepLabV3, PSPNet, PSANet, SegFormer and SETR, the proposed VTC-Net demonstrates notably superior performance in segmenting multi-granular coal particles. The image simultaneously displays multi-scale raw coal samples, with distinct variations in scale. Red rectangular boxes highlight regions where undersegmentation commonly occurs, while blue circular boxes indicate areas prone to misclassification. Within the circular regions, VTC-Net achieves nearly error free identification and significantly reduces undersegmentation in the rectangular areas. For the segmentation of multi-grain-size ore (Images 1–4), VTC-Net maintains robust and consistent performance across varying grain scales, substantially mitigating errors in fine coal classification and excessive fusion of coarse coal. For coal particles with similar sizes and adhering edges (Images 1–4), the four benchmark models still exhibit noticeable errors, including residual adhesion between particles, misclassified regions, and merged segments of different sizes. In contrast, VTC-Net maintains high segmentation accuracy in most cases and effectively separates adhered particle boundaries.

Beyond the annotated regions, VTC-Net also shows stronger capability in recognizing multi-granular particles, with lower misclassification rates and more precise boundary delineation. While other models display partial errors or undersegmentation, VTC-Net accurately outlines particle contours and distinguishes adhesive interfaces clearly. The VTC-Net model demonstrates superior performance in segmentation scenarios characterized by coexisting multi-scale variations and adhesions. These results confirm VTC-Net’s ability to overcome challenges posed by complex backgrounds and substantially improve edge segmentation precision.

### 4.5. Feature Visualization and Analysis

**Figure 6:**
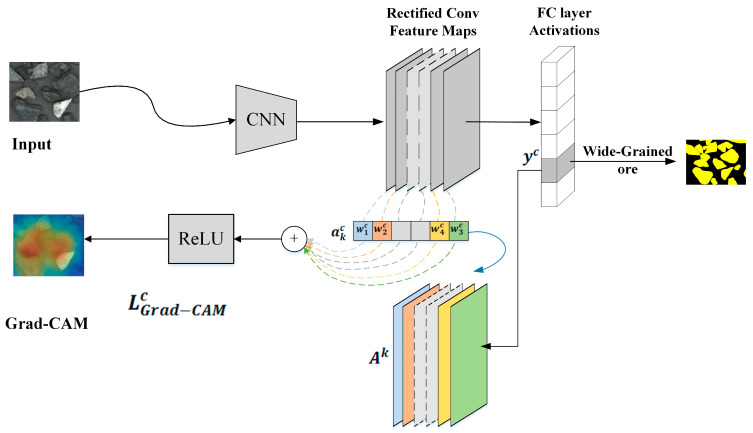
Structure diagram of the Grad-CAM network.

Feature visualization is essential for analyzing the key regions on which a model focuses and for guiding model optimization. Currently, mainstream visualization methods include Feature Map, CAM, and Grad-CAM [46]. Compared to the first two, Grad CAM does not require architectural modifications and offers stronger generalization, making it the primary choice for complex scenarios. The structure of the Grad-CAM network is shown in Figure 6. In challenging conditions such as particle adhesion or stacking, the Grad-CAM algorithm utilizes gradient information to capture the model’s priority of attention toward adhering ore particles. This further enables the analysis of differences in the network’s response intensity at ore boundary regions, helping to understand why the model makes certain predictions in ore segmentation tasks.

**Figure 7:**
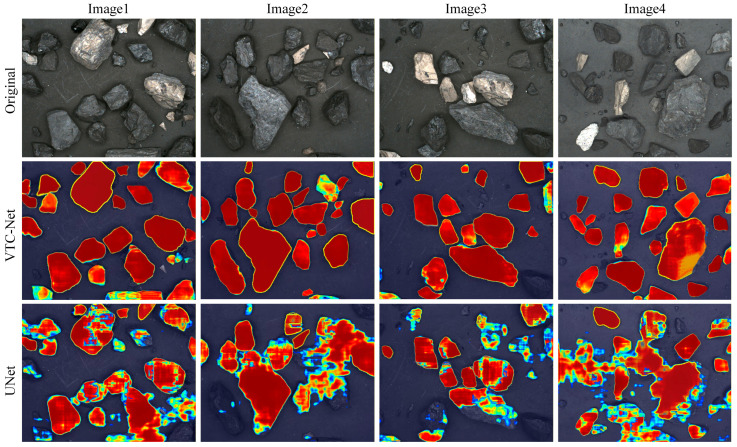
The Grad-CAM result graphs of VTC-Net and the baseline network UNet.

Figure 7 displays the Grad-CAM results of VTC-Net and the baseline UNet network. Red indicates high activation in the region, while blue represents weak activation in the predicted category. The analysis reveals that the classical UNet model struggles with accurate positioning of raw coal targets, particularly in effectively identifying and segmenting adhered coal particles. In contrast, VTC-Net demonstrates significantly larger active regions for coal particles, achieving more precise alignment with their actual contours and showing heightened focus on adhered coal particles. Therefore, the proposed VTC-Net outperforms the baseline in segmenting adhered coal particles.

## 5. Conclusions

To address the semantic segmentation challenges in mineral processing caused by multi-scale particle coexistence and particle adhesion, this study proposes a VTC-Net segmentation model that integrates Transformer, CBAM attention mechanism, and BatchNorm. Through systematic experimental analysis, the key findings are as follows:

(1) The VTC-Net model effectively mitigates the common issues of “undersegmentation” and “misjudgment” in traditional methods for complex raw coal images. It achieves optimal segmentation performance (MIoU of 89.90%) on the validation set, significantly outperforming multiple classical segmentation networks. This demonstrates the architecture’s distinct advantage in enhancing segmentation accuracy for multi-scale, highly cohesive coal particles.
(2) Ablation experiments demonstrate that the introduced Transformer module, CBAM module, and BatchNorm layer all significantly enhance model performance. The Transformer’s global context modeling capability and CBAM’s channel-space dual attention mechanism complement each other synergistically, enabling a more comprehensive capture of both long-range dependencies between raw coal samples and local feature details.
(3) The placement of the CBAM module significantly impacts performance. Its optimal placement is at the deep encoder layer (Feat4), where the feature carries richer semantic information, enabling the attention mechanism to more precisely enhance the raw coal target region and weak boundaries.
(4) The feature visualization (Grad-CAM) results demonstrate that, compared to the baseline model, the active regions of VTC-Net align more closely with the actual raw coal contours, with heightened focus on adhered areas, which intuitively explains the performance improvement.

In conclusion, the proposed VTC-Net model provides a high-precision inspection solution for online ore particle size analysis, offering practical value for advancing intelligent detection and control in mineral processing. Although the data augmentation strategies employed in this paper simulate common imaging conditions such as lighting variations and viewpoint changes to a certain extent, they still struggle to fully account for the complex factors that may exist in real industrial scenarios. Therefore, future work will focus on: (1) Collecting industrial data across diverse scenarios and ore types—including dust occlusion, severe motion blur, and intense camera shake—while expanding datasets with industry-specific augmentation techniques (e.g., simulating dust or mist) to enhance model generalization in complex environments; (2) Further reducing model complexity and accelerating inference speed to integrate the proposed segmentation model into actual ore processing and online inspection systems. This will enable field deployment and real-time performance validation, advancing the method toward engineering implementation.

## Figures and Tables

**Table 1:** Model training hyperparameters.

Parameters	Values	Parameters	Values
Input image size	512	Optimizer	Adam
Epochs	100	$\beta_{1}$	0.9
Batch size	8	Learning Rate	1 × 10^−4^

**Table 2:** Cross-Validation Experimental Results.

Fold	MIoU (%)	MPA (%)	Acc (%)
Fold1	85.48	87.48	96.44
Fold2	84.82	86.82	96.27
Fold3	83.55	85.95	95.98
Fold4	84.95	86.95	96.21
Fold5	84.54	85.54	96.20
Average	84.67	86.55	96.22
Standard deviation	0.64	0.70	0.15

**Table 3:** Comparison results with test set experiments.

Dataset	MIoU (%)	MPA (%)	Acc (%)
Fold1	85.48	87.48	96.44
Test Set	86.02	88.21	97.43

**Table 4:** Effects of CBAM addition position on the model.

Add Location	Feat1	Feat2	Feat3	Feat4	MIoU (%)	MPA (%)	Acc (%)
1	√				85.80	92.91	95.90
2		√			85.95	92.53	95.62
3			√		87.60	93.47	96.14
4				√	88.30	93.88	96.35
5		√	√		87.61	93.41	96.13
6		√		√	88.01	93.69	96.25
7			√	√	88.21	93.85	96.32
8		√	√	√	87.98	93.64	96.25
9	√	√	√	√	88.11	93.72	96.29

Note: 1 is to add CBAM only to Feat1; 2 is to add CBAM only to Feat2; 3 is to add CBAM only to Feat3; 4 is to add CBAM only to Feat4; 5 is to add CBAM to both Feat2 and Feat3; 6 is to add CBAM to both Feat2 and Feat4; 7 is to add CBAM to both Feat3 and Feat4; 8 is to add CBAM to both Feat2, Feat3 and Feat4; 9 is to add CBAM to both Feat1, Feat2, Feat3 and Feat4.

**Table 5:** Comparison of different attention mechanisms.

Models	MIoU (%)	MPA (%)	Acc (%)
UNet + SE	87.15	93.27	96.00
UNet + ECA	87.08	93.12	95.98
UNet + CA	88.01	93.64	96.26
UNet + CBAM	88.30	93.88	96.35

**Table 6:** Comparison of ablation experiment results.

Models	MIoU (%)	MPA (%)	Acc (%)
UNet	86.35	92.67	95.76
UNet + BatchNorm	87.43	93.39	96.12
UNet + Transformer	88.64	94.34	96.42
UNet + CBAM	88.30	93.88	96.35
UNet + Transformer + CBAM	88.73	94.18	96.46
VTC-Net	89.90	94.78	96.80

**Table 7:** Performance Comparison of VTC-Net with Other Mainstream Networks on Multi-granularity raw coal Datasets.

Models	MIoU (%)	MPA (%)	Acc (%)	Params	GFLOPs	Inference Speed (ms)
UNet	86.35	92.67	95.76	31.23	220.72	60.28
DeepLabV3	82.81	90.61	94.23	36.07	100.88	23.50
PSPNet	85.36	91.78	94.74	49.13	94.41	30.30
PSANet	85.33	91.94	94.75	39.69	55.69	25.06
SegFormer	82.49	87.79	92.55	3.72	6.78	19.78
SETR	68.08	77.71	88.60	64.56	230.78	69.58
VTC-Net	89.90	94.78	96.80	50.14	225.47	64.54

## Data Availability

The data presented in this study are available on request from the corresponding author. The data are not publicly available due to ongoing study.
